# Qualitative Methods in Pharmacy Research

**DOI:** 10.3390/pharmacy6030079

**Published:** 2018-08-02

**Authors:** Gisselle Gallego, Lotte Stig Nørgaard

**Affiliations:** 1 School of Medicine, The University of Notre Dame, Australia, Darlinghurst, NSW 2010, Australia; 2 Faculty of Health and Medical Science, Department of Pharmacy, University of Copenhagen, Copenhagen 2100, Denmark; lotte.norgaard@sund.ku.dk

**Keywords:** qualitative methods, pharmacy practice, research

## Abstract

Over the past years, there has been an increase in the use of qualitative methods in health services research, including pharmacy research. Pharmacy practice researchers can use these methods to understand, explain, discover, and explore both patients’ and health care practitioners’ thoughts, perceptions, and feelings. Qualitative research can also be used for the “democratisation” of research methods through research that is inclusive, collaborative, and involves partnerships and co-production. There is a wide spectrum of qualitative research methods that might be used in pharmacy research. This Special Issue showcases five articles in different settings and countries with diverse participants that seek to develop, explore, describe, and identify. These articles provide further insights into important pharmacy questions with the ultimate goal of helping improve health and well-being.

Over the past years, there has been an increase in the use of qualitative methods in health services research, including pharmacy research. Pharmacy practice researchers can use these methods to understand, explain, discover, and explore both patients’ and health care practitioners’ thoughts, perceptions, and feelings. Qualitative methods seem appropriate if we wish to learn from participants and the ways that they experience a process. It helps uncover and describe participants’ perspectives on a certain event. Qualitative research can also be used for the “democratisation” of research methods through research that is inclusive, collaborative, and involves partnerships and co-production. It can also provide a framework for research that is not only about or on participants, but rather with and by participants as co-creators [1]. There is a wide spectrum of qualitative research methods that might be used in pharmacy research, some of which are being showcased in this Special Issue of Pharmacy.

Santina et al. [2] used qualitative research as part of a mapping process to design a community-based pharmacy intervention. The authors conducted three descriptive exploratory qualitative studies that included different stakeholders and different data collection methods (individual interviews and focus groups). These data informed an Intervention Mapping (IM) process to design a community pharmacy-based intervention to optimise patients’ use of antidepressants [2]. This protocol outlined the steps involved in an IM process. Latif et al. [3] also used qualitative methods to co-develop a community-based digital educational intervention for marginalised communities. In this case, qualitative research gave a “voice” to vulnerable patients. It helped uncover their perspectives on pharmacy services and how they could be improved. The study highlighted the importance of listening to those who do not usually have a voice, and tailoring services to individual’s circumstances and needs without stigmatising or further marginalising vulnerable groups. Like Satina’s study, the study by Latif et al. used qualitative data to inform the design of an e-learning intervention for community pharmacists. Similarly, Humphries et al. [4] used qualitative methods to develop a community pharmacy intervention to improve adherence to endocrine therapy for breast cancer. These studies highlighted the importance of exploring patients’ views and identifying the potential barriers to community pharmacy-based interventions.

While the previous articles focused on a range of stakeholders, including patients and consumers, two other studies focused on health care practitioners. Wood et al. [5] explored the barriers and facilitators to the implementation of Chlamydia partner treatment in Western Australia from the providers’ perspective. This qualitative study involved interviews with health care professionals involved in standard therapy (general practitioners, nurse practitioners, and sexual health clinicians) and community pharmacists. The goal of this study was to inform an effective alternative pathway for partner treatment of Chlamydia. Croft et al. [6] used the “think-aloud method”, which is often used to investigate problem solving and commonly used in cognitive psychology research, to investigate pharmacists’ clinical reasoning and the decision-making process that is used when supplying prescribed medicines. This qualitative study described the pharmacist decision-making process and provided insights into the clinical reasoning process. Pharmacy educators can replicate this study to understand the gaps in knowledge and implement educational interventions to improve this process. 

The articles in this Special Issue have illustrated that qualitative research offers unique opportunities for understanding complex phenomena such as marginalised communities’ medication-taking experiences. It also provides a better understanding of stakeholders’ experiences and a catalyst for further work. It can be used as part of a mixed methods project to provide insights and complement quantitative data. Last but not least, it encourages research that is concerned with ensuring that participants who experience marginalisation influence research, help identify what is important, and specify how the community might benefit from their involvement. We hope this Special Issue will motivate other researchers to use these methods to provide further insights into important pharmacy questions with the ultimate goal of helping improve health and well-being.
